# Understanding the effects of mild traumatic brain injury on the pupillary light reflex

**DOI:** 10.2217/cnc-2016-0029

**Published:** 2017-08-03

**Authors:** Kenneth J Ciuffreda, Nabin R Joshi, James Q Truong

**Affiliations:** 1 Brain Injury Research Clinic, SUNY/State College of Optometry, 33 West 42nd Street, New York City, NY 10036, USA

**Keywords:** mTBI, mild traumatic brain injury, photosensitivity, PLR, pupillary light reflex

## Abstract

The pupillary light reflex represents an optimal visual system to investigate and exploit in the mild traumatic brain injury (mTBI) population. Static and dynamic aspects of the pupillary light reflex were investigated objectively and quantitatively in the mTBI population. Pupillary responsivity was found to be significantly delayed, slowed and reduced, but symmetrical in nature, and with a smaller baseline diameter, as compared with normals. Several pupillary parameters also discriminated between those with versus without photosensitivity. Thus, dynamic pupillometry provides several objective biomarkers for the presence of mTBI and photosensitivity, gives insight into the global sites of neurological dysfunction and possible related mechanisms, and should result in improved patient care.

Some believe that the pupil is the window to the soul. The pupil may also be a window to mild traumatic brain injury (mTBI).

The area of mTBI has been in the forefront of the medical world for more than a decade. This was primarily as a result of the USA’s recent military encounters in Iraq and Afghanistan, where TBI was the ‘signature injury’ and frequently the ‘invisible injury’, as well as from the sports arena, in particular football with its potential link to chronic traumatic encephalopathy [1]. There are approximately 1.8 million mTBIs in the USA annually, primarily from motor vehicle accidents, falls and sports/recreational accidents [2], with perhaps up to 10 million worldwide [2]. The resultant injury to the brain and surrounding microenvironment, frequently being of a coup–contrecoup nature, produces widespread neural damage. This is especially the case for the white matter tracts, which become stretched/deformed and at times broken leading to neural signal processing errors, distortions and delays [2–4]. In addition, this pervasive brain injury results in a constellation of general medical problems of a sensory, motor, perceptual, cognitive, attentional, physical, physiological and/or behavioral nature [2–5]. For example, there may be problems with impulse control, sleep, attention and memory, to name a few. More specifically, it may produce a constellation of visual problems of a sensory (e.g., reduced contrast sensitivity, visual field deficits), motor (e.g., vergence dysfunction, saccadic dysmetria) and/or perceptual (e.g., impaired distance perception, difficulty with figure–ground discrimination) nature [2–6]. However, the visual dysfunction relevant to the present review is that of the pupillary light reflex (PLR).

The pupils are routinely studied by clinicians to assess, in part, the neural integrity of the visual system. Clinically, abnormal pupil size and responsivity provide important clues to detect the site and nature of various lesions along its extensive afferent and efferent pathways. Recently, with the availability of modern pupillometers, the clinician and researcher now have the ability to assess subtle abnormalities in steady-state pupil size and dynamics of the direct and consensual pupillary responses.

In performing the review, Pubmed Central/National Library of Medicine databases were searched for pertinent articles using any combination of the terms: mild traumatic brain injury, mTBI, concussion, pupil, pupillometry, infrared pupillometry, blast versus blunt injury, pediatric mTBI, vision deficits, photosensitivity and other appropriate keywords discussed under the various headings in the paper. Google scholar and the SUNY College of Optometry library resources were also searched in a similar manner. All of the selected articles were published in peer-reviewed journals, and public doctorate dissertations were also cited when appropriate. Only original articles published in English were included, and books from renowned researchers in their respective fields were also referenced.

As we shall see, the PLR in mTBI is delayed, slowed, reduced and symmetrical in its responsivity.

## The PLR pathway

The pupil is approximately a circular aperture positioned slightly inferiorly and nasally with respect to the center of the cornea. Optically, the pupil helps in reducing various imperfections (e.g., aberrations) of the eye, which in effect improves the range of clear vision by increasing its depth-of-focus. The pupil is controlled by two distinct sets of smooth muscles. The circular muscles surrounding the pupillary aperture are innervated by the parasympathetic pathway of the autonomic nervous system (ANS), and they act to constrict the pupil. In contrast, the radial muscles surrounding the pupillary aperture are innervated by the sympathetic pathway of the ANS, and they act to dilate the pupil. The interaction of these two muscle groups gives rise to the pupillary reflex. The PLR is a visual reflex that regulates the diameter of the pupil, and hence controls the amount of light impinging upon the retina. It is driven chiefly by the luminous intensity of the incoming light, but it can be influenced by other factors, such as attention, drugs, visual adaptation level, emotional state and many diseases [7–9].

The ANS innervates and controls the size of the human pupil over a very wide range of 12 log units of light intensity [8]. Light passing through the pupil is absorbed by the photopigments in the retina. The rods and cones comprise 98% of photoreceptors, while the intrinsically photosensitive retinal ganglion cells (ipRGCs) comprise the remainder, with each receptor participating in the PLR. The rods and cones primarily participate in the early phases of the response, whereas the ipRGCs primarily participate in the latter ‘sustained’ constriction response phase [9]. Rods and cones contain rhodopsin and iodopsin as their photopigment, respectively, while the ipRGCs incorporate melanopsin.

As the nerve fibers from the retina travel toward the thalamus, there is decussation of the nasal pupillary and visual fibers. This establishes the anatomical basis for the consensual reaction between the pupils. The majority of the axons of the retinal cells transmitting light information next travel to the lateral geniculate nucleus in the thalamus, while a minority of axons is directed to the hypothalamus and the olivary pretectal nucleus (OPN).

**Figure 1.**
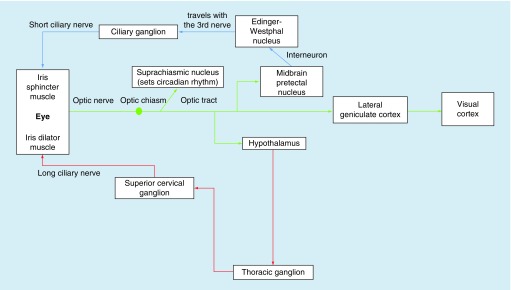
**Schematic of the afferent and efferent arms of the sympathetic and parasympathetic neural pathways circuitry of the pupillary light reflex.** Reprinted with permission from [11]

From the OPN, the fibers run along two distinct pathways: the parasympathetic and sympathetic, with both systems sharing and responding to the light input from the retina. The parasympathetic pathway comprises a 4-neuron arc: the first involves the light input from the retina to the OPN; then the nerve fibers from the OPN (i.e., interneurons) travel (both crossed and uncrossed) to the Edinger–Westphal (EW) nucleus in the midbrain; these fibers then course from the EW to the ciliary ganglion (preganglionic fibers); lastly, from the ciliary ganglion, the postganglionic fibers travel to the iris sphincter muscles via the short ciliary nerve, thus resulting in pupillary constriction. Similarly, the sympathetic system is also comprised a 4-neuron circuit: the first transmits the visual light input from the retina to the OPN; the second carries information from the hypothalamus down the brainstem (uncrossed) along the spinal cord to the cervicothoracic level of C7–T2 (central neurons); then, the preganglionic neurons synapse from the cervicothoracic level to the superior cervical ganglion at the level of the carotid artery bifurcation; lastly, the postganglionic fibers travel from the superior cervical ganglion to the iris dilator muscles via the ciliary nerves, thus resulting in pupillary dilation [10,11]. A concise schematic representation of the pupillary pathway is presented in Figure 1 [11].

Thus, given the above neuroanatomy, the PLR pathway is relatively complex. Damage anywhere along its route can result in abnormal static (i.e., steady-state or baseline) and dynamic (i.e., transient) pupillary responsivity. This is certainly the case in mTBI with its frequent and pervasive coup–contrecoup insult and related diffuse axonal injury type of brain damage.

## Review of normal static & dynamic pupillary light responses

### Static aspects

There are several factors that need to be considered and briefly discussed here for general knowledge, as well as for later comparison with the mTBI findings [7–8,10–12]:

- The pupillary diameter under standard clinical test conditions typically ranges from approximately 2 to 5 mm in adults;
- The average age-related decrease in pupillary diameter is 0.3 mm/decade, likely due to iris stiffening;
- In addition to age, other factors that may influence pupillary diameter are emotional state, attention, fatigue, level of light adaptation, sleep, iris color and refractive state, among others;
- Under steady-state conditions, a small amount of pupillary unrest/oscillations is evident, likely due to pharmacological and neurological fluctuations in the parasympathetic and sympathetic innervational pathways and their interactions; interestingly, either the presence of markedly reduced or increased ‘unrest’ is suspect for neurological insult/abnormality;
- In addition, it is estimated clinically that 4% of the general population has anisocoria of greater than 1 mm, and hence are suspect for neurological disease; in contrast, normal physiological anisocoria is found in approximately 17% of the general population;
- The pupillary system exhibits monocular, area-based light summation, as well binocular light summation;
- The pupillary system also exhibits temporal summation;
- The pupillary response amplitude increases with increase in light intensity, light duration and a greater dark-adaptation level;
- The initial pupillary constriction can reduce the level of retinal illumination by up to 1.5 log units, thus providing an immediate mechanism for partial light adaptation in the first 500–1000 ms, with subsequent longer term light adaptation occurring at higher level neural sites;
- Since the eye's foveal area is more sensitive than the retinal periphery to changes in light intensity, stimulation of the former will produce a greater response than to the latter region;
- The pupillary response exhibits chromatic spectral sensitivity.

### Dynamic aspects

There are several factors that need to be considered and briefly discussed here for general knowledge, as well as for later comparison with the mTBI findings [8,10–15]:

- Pupillary latency (i.e., reaction time, RT) progressively decreases with an increase in stimulus light intensity, with a range from approximately 500 to 200 ms;
- Pupillary latency increases approximately 1 ms/year, as per other age-related RT measures;
- Pupillary dynamic response amplitude (i.e., change) increases with increase in stimulus light intensity, with a typical dynamic range of approximately 0.25–2.5 mm;
- Pupillary maximum/peak constriction velocity increases with an increase in stimulus light intensity, with a range of approximately 0.5–7.5 mm/s; this peak/average velocity–response amplitude relationship has been termed as the main sequence [11,15], and it reflects midbrain responsivity and integrity;
- Pupillary maximum/peak dilation velocity (PDV) increases with an increase in stimulus light intensity (i.e., per the above ‘main sequence’ relation), with a range of approximately 0.25–2.5 mm/s;
- Thus, peak constriction velocity is approximately two- to three-times faster than PDV; the same is true for average constriction versus average dilation velocity, but as expected the values here are considerably smaller than for peak velocity;
- Overall the majority of the pupillary response time is typically approximately 6 s, with about 1 s for the initial constriction dynamic phase and about 5 s for the subsequent dynamic dilation phase;
- Dynamic constriction responses are consensual between the two pupils;
- Responses are nonlinear (i.e., a range nonlinearity), being greatest when the baseline pupillary diameter is 4.0–5.5 mm, thus likely reflecting an optimal biomechanically based response condition;
- The parasympathetic system primarily drives pupillary constriction, whereas the sympathetic system primarily drives pupillary dilation.

## Static & dynamic pupillary light responses in mTBI: test protocols & instrumentation

There has been a paucity of investigations into the PLR in humans with mTBI. In one group headed by Capo-Aponte, the Neuroptics [16] hand-held, monocular, infrared, objectively based pupillometer (PLR-200) was used having only one, fixed, nonpatterned flash stimulus configuration available (180 microwatts, 167 ms duration) [13]. See Supplementary Figure 1A. The PLR was assessed in adults (n = 20) in the military in their subacute phase (15–45 days post injury) of mTBI due to blast injury, with control-matched comparisons (n = 20). In the other research group, headed by Ciuffreda, either the above monocular pupillometer was used in an adult group in their chronic phase (>45 days post insult) of mTBI (n = 17) and with 15 normal controls [14], or the Neuroptics binocular (DP-2000) was employed with either binocular recording and binocular stimulation or binocular pupillometer recording and monocular stimulation [17–20], with a full range of nonpatterned, flash stimulus characteristics available (see Supplementary Figure 1B). These stimuli now included the following: either 100 ms (pulse) or 1000 ms (step) test durations; either 4 lux (dim) or 251 lux (bright) light levels; and either white, blue or red flashes, all performed in subdued room illumination (5 lux) with pretest visual adaptation of 10 min. In the latter study, the PLR was assessed in adults with mTBI (n = 32) in the chronic phase (i.e., greater than 45 days post injury) of the nonblast injury nature (typically motor vehicles accidents, sports and falls), with age-matched controls (n = 40). None had evidence of an afferent pupillary defect (APD) per the clinical swinging flashlight test [7]. Both recording systems have a resolution of 0.05 mm with a sampling rate of 30 Hertz (i.e., 30 samples/s).

For the monocular studies [13,14], the pupillometer's software automatically measured and quantitatively analyzed the following seven parameters: baseline pupil diameter (i.e., steady-state diameter before the light stimulus is presented), minimum pupil diameter (i.e., diameter at maximum constriction following the stimulus presentation), response amplitude (i.e., the difference between the maximum and minimum pupillary diameters), response latency (i.e., the time between the stimulus onset and initiation of the constriction response; RT), mean (or average) constriction and dilation velocities and maximum (or peak) constriction velocity.

**Figure 2.**
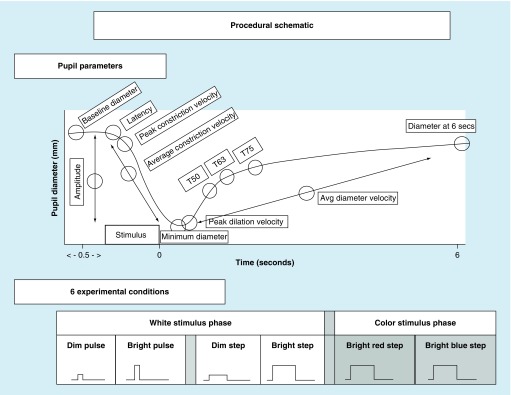
**Dynamic pupillary response profile and stimulus conditions.** Top: Schematic representation of a pupil response profile and the associated pupil parameters assessed as indicated by the open circles. The prestimulus time is 0.5 s, and the post-stimulus time is 6.0 s. Bottom: Schematic representation of the six possible experimental test stimulus conditions. The x-axis represents the relative time, and the y-axis represents the relative stimulus intensity. Dim = 4 lux, bright = 251 lux, pulse = 100 ms and step = 1000 ms. Reprinted with permission from [11].

In addition, the two pupillometers also provided a raw numeric data stream of pupillary diameter over time, thus allowing for calculations of nonstandard/more unique parameters as needed for the specific study requirements. Hence, for the four binocular studies [11,17–20], in addition to the aforementioned parameters, the following additional parameters were assessed: PDV, the 6 s poststimulus pupil diameter (6PSPD) and redilation recovery times (i.e., T50-, T63- and T75-times, that is the time to recover/redilate to 50, 63 and 75% of the initial baseline diameter, respectively); see Figure 2.

In the following section, the static and dynamic aspects of the human pupillary response in mTBI, with comparison to visually normal (VN) individuals, will be reviewed and discussed in the context of four key questions.

### Question 1: what is the effect of mTBI on static & dynamic interocular pupillary asymmetry?

There have not been any investigations assessing dynamic pupillary asymmetry objectively in normals. However, there have been two primary studies dealing with static pupillary asymmetries under baseline conditions in the dark in normals, as described below.

In the first study [21], the main objective was to characterize the normal upper limit of the static interocular pupillary asymmetry (IOPA; i.e., any asymmetry of the two pupils in their baseline state). Infrared photography was used to obtain simultaneous images of the two pupils in the dark in 425 children and 425 older adults. The majority (∼75%) exhibited static asymmetry of ≤0.3 mm. Thus, about 25% had some additional degree asymmetry of up to 1.2 mm. The author hypothesized that presence of asymmetry stemmed from normal interocular variation of central neuronal control (i.e., neural noise-related asymmetries of the supranuclear inhibitory control of the EW nucleus). Thus, Loewenfeld coined the term ‘simple central anisocoria’. However, she did not calculate a precise average for this asymmetry within her normal population sample, but rather only identified an upper limit cut-off for ‘normalcy’.

A later paper was by Lam *et al.* [22]. Their objective was to determine the prevalence of simple anisocoria in the general healthy population (n = 128; ages 8–92 years). Infrared photography was again used to obtain simultaneous images of the two pupils in darkness. Using the criterion of 0.4 mm or greater pupillary asymmetry, which reflected the minimum magnitude of asymmetry that they believed is ‘readily visible’ by the unaided eye of the clinician, they concluded that the prevalence of anisocoria was approximately 19% in the general normal population, similar to that found by Loewenfeld (25%) [21], but with a maximum difference of 0.6 mm versus the value of 1.2 mm in the Loewenfeld study [21]. However, again a precise average of the static asymmetry within the population was again not calculated.

Given the above relative paucity of information on this important topic in normals, and none in the mTBI population, Truong and Ciuffreda [18] investigated both static and dynamic pupillary asymmetries in both populations. The pupils of both eyes were assessed simultaneously following 10 min of visual adaptation in a dark room environment (5 lux room illumination). The test mode was light stimulation to only one eye with binocular recording of the pupillary responses. See earlier description in section ‘Static & dynamic pupillary light responses in mTBI: test protocols & instrumentation’.

Static IOPA was defined as the steady-state difference in pupillary diameter between the two eyes under constant low illumination conditions. In the normal population, the average IOPA was 0.26 ± 0.20 mm, or 3.93 ± 3.03%. In the mTBI population, the average was 0.26 ± 0.19 mm, or 4.48 ± 3.57%. While the mTBI average was numerically larger in percentage, it was not statistically different than that found in the normal group (p > 0.05). Thus, combining the two groups together for a better global parameter estimate, the average static IOPA was 0.26 ± 0.20 mm, or 4.17 ± 3.29%.

Dynamic IOPA was defined as the difference in response amplitude of constriction (i.e., initial baseline minus minimum pupillary diameter) following monocular light stimulation (with binocular recording). The data were assessed for each of the four white light conditions (dim pulse and step, bright pulse and step), as well as the combined average across all four stimulus conditions. The dynamic IOPA was not statistically different between the two groups (p > 0.05) under any of the test conditions. Thus, for a better global parameter estimate, the data from the two groups were combined for each of the four test conditions. The largest average dynamic asymmetry of 0.16 mm, or 2.67%, occurred under the dim step condition (i.e., 4 lux, 1000 ms), while the smallest average dynamic asymmetry of 0.09 mm, or 1.36%, occurred under the bright step condition (251 lux, 1000 ms). Lastly, when the data across all four test conditions were combined across both groups, the ‘grand’ average dynamic interocular asymmetry was 0.11 mm, or 1.84%, with a standard deviation of 0.10 mm, or 1.70%.

While the presence of mTBI typically adversely affects many aspects of the dynamics of the PLR [11,13–14,17–20], these effects appear to be symmetrical rather than asymmetrical in nature. That is, a mTBI *per se* typically does not cause asymmetric pupillary damage and related asymmetric responsivity. Furthermore, the present findings show that most mTBI patients exhibited negligible pupillary asymmetry (i.e., either static anisocoria or a difference in dynamic constriction amplitude), with this amount being similar numerically and statistically to that found in the normal population.

These findings provide an important clinical insight. The largest degree of pupillary asymmetry was found with the dim-step test condition, which is not used in routine clinical screening of pupillary responsivity; rather a bright, prolonged (i.e., a few seconds) step of illumination is the norm [7–8,12]. This area needs further investigation to determine the precise stimulus test condition for diagnostic optimization of pupillary asymmetry in both the general and neurological clinic populations. However, these findings are consistent with conventional clinical assessment for an APD, which typically employs a minimum of a 0.3 neutral density filter (50% reduction in light transmission) to ‘balance’ the pupils with respect to the initial pupillary asymmetry, thus likely reflecting an afferent, neurologically based deficit [7–8,12].

From a physiological and anatomical perspective, these results are logical. Since the majority of the neural circuitry of the PLR is the same in both hemispheres of the brain [12], it follows that both the direct and consensual responses of the PLR would typically be relatively symmetrical. The small section of neural circuitry that is exclusive to one hemisphere is the region anterior to the optic chiasm (i.e., the section from the retina to the optic chiasm). At the chiasm, approximately 50% of the pupillary fibers decussate, and thus any defect or damage posterior to the chiasm should affect both pupils equally. Since baseline pupillary diameter is primarily a function of the sympathetic system, and its primary innervation is the EW nucleus, which is all located postchiasmally, it is not logical for most diffuse head trauma alone to cause an increase in pupillary asymmetry *per se*. Indeed, this is what was found. Postchiasmally, the circuitry of the PLR is the same on both hemispheres of the brain, and thus any defect posterior to the chiasm should affect both pupils equally.

### Question 2: what is the effect of mTBI on the dynamic PLR?

As described earlier, there have been two prior studies dealing with dynamic pupillary responsivity in mTBI [13,14]. However, the results were equivocal.

In the Capo-Aponte *et al.* investigation [13], the following parameters were found to be significantly different (p < 0.05) between the two groups, all of which were slower/delayed in the mTBI group as compared with the VN cohort:

- Average constriction velocity;
- Average dilation velocity;
- Constriction latency;
- T75 dilation recovery.

In the later Thiagarajan and Ciuffreda study [14], the following parameters were found to be significantly different (p < 0.05) between the two groups, all of which were slower/delayed/smaller in the mTBI groups as compared with the VN group:

- Average constriction velocity;
- Average dilation velocity;
- Maximum constriction velocity;
- Maximum pupil diameter;
- Average constriction amplitude.

Thus, agreement was only found for the first two parameters listed above despite using the same automated test device. There are two likely reasons for these differences. In the former investigation, subjects were all tested in the subacute phase of their insult, and furthermore all had blast-induced head injuries. In the latter study, however, subjects were all tested in the chronic phase, and furthermore none were blast induced. This suggests that testing at each of the three phases of insult, namely the acute, subacute and chronic phases may be necessary and informative in the mTBI population to determine the specific phase-dependent, diagnostic, dynamic pupillary abnormalities. Further work here is warranted, especially in the extremely early acute phase, for example, minutes later at the football sideline, as well as hours later after a suspected insult.

Due to the above population differences and the relatively small sample sizes in the above studies, a larger and more comprehensive investigation was more recently performed [11,17–20]. This included binocular stimulation and binocular recording, larger sample sizes (n = 32 mTBI; n = 40 VN), more test stimulus parameters/conditions (6 vs 1, namely white dim pulse and step, white bright pulse and step, bright red [622 nm] step and bright blue [463 nm] step), additional analytical procedures (i.e., ‘main sequence’ [15] and receiver operating characteristics [ROC] analysis [23]), and the assessment of more dynamic pupillary parameters (9 vs 8).

The different chromatic (colored) test stimuli were used to differentiate between the three classes of photoreceptors: white light primarily stimulated the rods and cones, with only a very small/weak contribution from the extreme paucity of ipRGC cells (i.e., 98 vs 2%); red light stimulated the rods and cones only, with virtually no ipRGC/melanopsin stimulation/contribution; and blue light stimulated the rods and cones, but with a very large/strong, ipRGC/melanopsin contribution as their spectral sensitivity is blue dominated [9]. The populations were the same as described earlier in section ‘Static & dynamic pupillary light responses in mTBI: test protocols & instrumentation'.

**Figure 3.**
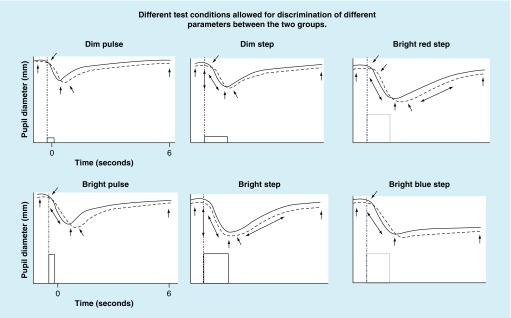
**Schematic representation of pupillary response profiles for the six test conditions and two diagnostic groups.** The solid lines represent the typical normal response, and the broken lines represent the typical mild traumatic brain injury response for each of the six test conditions. The arrows depict the abnormal parameters found in the mild traumatic brain injury group as compared statistically to the normal group (p < 0.05). Reprinted with permission from [11]

The findings are summarized schematically in Figure 3 for the six test conditions and two diagnostic groups. The arrows depict those parameters that significantly differentiated between the two groups (see Figure 2 for detailed specification of parameters):

- The dim white pulse condition had the least number of pupillary parameters that differentiated between the two groups (five out of nine), whereas the bright red step condition had the greatest number of parameters (eight out of nine; only constriction amplitude did not).
- The dim white step and the bright white step, with the latter coming closest to what is done clinically, also differentiated at a high level (seven out of nine; only peak constriction velocity and constriction latency did not for the latter condition).
- In general, the mTBI cohort exhibited longer constriction latency times, slower velocities and smaller pupil diameters (baseline, minimum and 6PSPD).
- Several pupillary parameters discriminated between the two groups for all six test conditions: maximum and minimum diameter, average constriction velocity, PDV and the diameter at 6PSPD.

**Figure 4.**
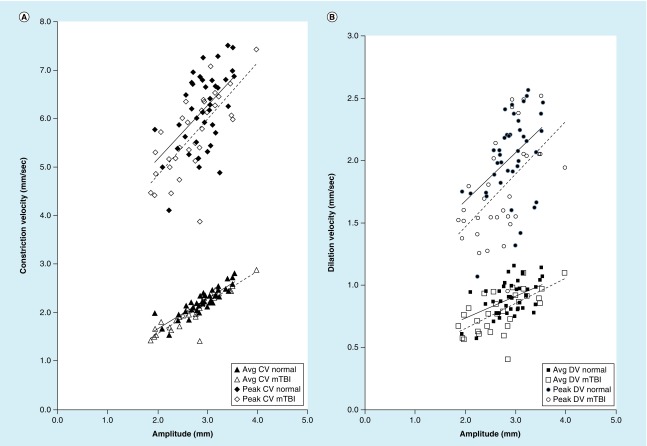
**Main sequence relation.** **(A)** The main sequence for peak constriction velocity (top), and average constriction velocity (bottom), under the optimal test stimulus condition (bright red step), **(B)** the main sequence for peak dilation velocity (top) and average dilation velocity (bottom), under the optimal test stimulus condition (bright red step). The solid line represents the normal group, and the broken line represents the mTBI group. All four velocity parameters were significantly different (p < 0.05) under this condition, being slower in the mTBI group. Avg CV: Average constriction velocity; Avg DV: Average dilation velocity; CV: Constriction velocity; DV: Dilation velocity; mTBI: Mild traumatic brain injury. Reprinted with permission from [11]

The ‘main sequence’ amplitude/velocity relation also clearly delineated between the two groups. There are three important points. First, as expected and mentioned earlier, both the peak and average velocity increased with increase in response amplitude, for both constriction and dilation, which reflects the underlying midbrain responsivity (Figure 4). Second, peak velocity for the VN group, on average, was significantly above the mTBI group for a given response amplitude, especially for the bright red step, for both constriction and dilation, thus suggesting abnormal midbrain responsivity and related damage in mTBI; this difference was not as pronounced for the average velocity parameter between the two groups (Figure 4). Third, the bright white step test condition for the PDV parameter best differentiated between the two groups, with this condition having the greatest offset between groups in linear regression fit (i.e., intercept value), and therefore best isolating the two diagnostic groups. Thus, this last test condition may serve as a good rapid, objective, diagnostic discriminator between these two groups.

The finding of significantly increased constriction latency in the mTBI group (194–214 ms across conditions) as compared with the normal group (182–199 ms across conditions) suggests an afferent-based pathway dysfunction in the former group. This was found to be the case in five out of the six test conditions, with the exception being the bright white step. Thus, the presence of a latency deficit was typically found, or at least most consistently uncovered, under the least intense stimulus condition. This is in line with the finding that a neurologically damaged visual pathway may exhibit exacerbated response abnormalities when the stimulus luminance in reduced [24,25]. Furthermore, and consistent with the above, with the most robust and intense stimulus condition (i.e., bright white step), response saturation may occur, and in effect mask any abnormal transient, subtle response differences.

The present findings also suggest presence of an efferent-based abnormality in both the parasympathetic and sympathetic systems. First, average constriction velocity was significantly reduced in the mTBI cohort, with this being parasympathetically driven. Interestingly, this was found for the five most intense stimulus conditions, but not for the least intense (i.e., the dim pulse). Hence, more stimulus drive to the pupillary system appears to be needed to differentiate between the two groups for this specific parameter. Second, several findings support the notion of an abnormal, sympathetic pupillary drive: maximum, minimum and the 6SPSD pupillary parameters were reduced in the mTBI group, as well as PDV [11].

**Figure 5.**
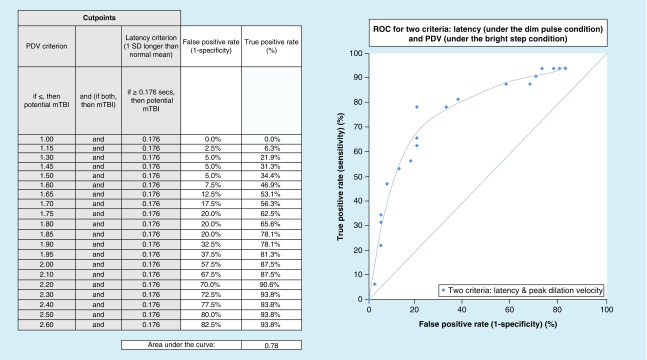
**Receiver operating characteristics curve for two selected parameters under different test conditions.** Cutpoints for peak dilation velocity and constriction latency were combined to produce this receiver operating characteristics curve. The latency cutpoint was set at 1 standard deviation longer than the normal mean, while the cutpoint for peak dilation velocity was varied. PDV: Peak dilation velocity; ROC: Receiver operating curve. Reprinted with permission from [11].

Lastly, ROC curves [23] were investigated with the hope of uncovering potential optimal, objective, diagnostic biomarkers for mTBI. ROC analysis, which is derived from signal detection theory, plots the true positive rate against the false positive rate for a range of different possible criteria in a specified diagnostic group. In essence, it graphically depicts the relation between sensitivity and specificity, with an increase in one accompanied by a decrease in the other. Values can range from 0.00 to 1.00, with higher values (e.g., 0.8) suggesting better predictive ability. One or more physiological system parameters can be incorporated to assess its predictability. This is shown in Figure 5, where two parameters were combined in the ROC assessment, namely pupillary latency and PDV, with a respectable value of 0.78. Thus, as shown in the adjacent table in Figure 5, for a PDV of 1.85 mm/s and a latency of 0.176 s, the true positive rate would be 78.1%, with a false positive rate of 20%, which is reasonable. Similar ROC analyses were also performed for the symptom of photosensitivity (PS) in both the normal and mTBI groups [19], with similar reasonable values obtained but with different critical parameters for each diagnostic group (e.g., 6SPSD in normals and dynamic constriction amplitude in mTBI). This suggests that with the combined use of selected, objectively based, pupillary parameters and the ROC analysis, one could now also have optimal, objective, diagnostic biomarkers for photosensitivity in both the mTBI and VN groups (see question 3 below), as well as for detecting the presence of mTBI.

### Question 3: is there a relation between the PLR & PS in mTBI?

One of the main vision symptoms reported by individuals with mTBI (and TBI in general) is PS [26]. This refers to visual discomfort in the presence of illumination levels and lighting sources (e.g., fluorescent lights) that are normally not very bothersome to others. Its prevalence in VN individuals is 10%, whereas it is approximately 50% in those with mTBI, in both the military and civilian populations [13,26]. Its ability to lessen and/or dissipate may have a long time-course, typically several months to several years [26], and at times not appearing to resolve at all. For example, within the first year following the brain insult, only 10% reported a reduction in their PS, while after more than 1 year post insult (up to 18 years), it was found that PS resolved in an additional 40% of symptomatic individuals [26]. Thus, it did not appear to resolve in the remaining 50%. In addition, the findings suggested that prescription of a dense (e.g., 80%) versus a less dense (e.g., 30%) gray or chromatic tint may be contraindicated, as a dense tint does not allow for much visual/neural adaptation to take place due to the markedly reduced amount of light entering the eye. Other factors that were related to lack of adaptation to PS were presence of hyperacusis, dry eye, migraines and loss of consciousness at the time of injury. However, the use of contact lenses appeared to promote adaptation to PS. Lastly, reduction and/or resolution of PS was not related to gender, age at injury, type of provocative illumination, number of brain injuries, refractive status, medications, types of therapy received, presence of other specific oculomotor dysfunctions (e.g., accommodative insufficiency) or presence/type of visual field deficit.

A link between PS and pupillary responsivity in this population has only recently been established using objective measures [19]. The subject populations and test conditions were the same as described earlier in ‘Static & dynamic pupillary light responses in mTBI: test protocols & instrumentation,’ with all 12 pupillary parameters assessed.

In the VN population with PS (6 out of the 40, or 15%), the following four pupillary parameters were significantly different as compared with their non-PS, normal cohort, when the data were combined across the white light and red light test conditions, as they gave similar response characteristics (p > 0.05), and hence were combined for greater power:

- Response amplitude;
- Average constriction velocity;
- Maximum constriction velocity;
- T50.

**Figure 6.**
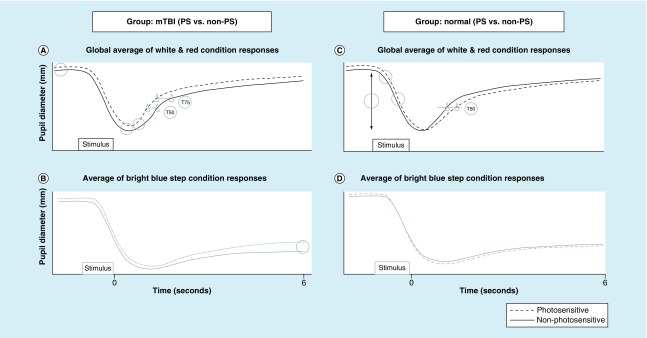
**Plotted is pupil diameter as a function of time.** **(A)** Schematic representation of the global average of the white and red condition pupil response for the mild traumatic brain injury group. **(B)** Schematic representation of the average bright blue step condition pupil responses for the mild traumatic brain injury group. **(C)** Schematic representation of the global average of the white and red condition pupil response for the normal group. **(D)** Schematic representation of the average bright blue step condition pupil responses for the normal group. The open circles indicate the statistically significant pupil parameters that differentiated those with versus without photosensitivity in both diagnostic groups. The triangles show the T50 and T75 response differences in the two profiles. Reprinted with permission from [11].

However, with the blue test light, none of the parameters differentiated those with versus without PS in the normal group; see Figure 6.

Thus, those with PS in the VN group exhibited more robust pupillary constriction, as reflected in their larger response amplitudes, related faster constriction velocities as per the main sequence relation, and more prolonged constriction time (i.e., a slower T50 redilation recovery time). See Figure 6.

In the mTBI population with PS (22 out of the 32, or 65%; about 4.5 times more prevalent than in the VN group), the following five pupillary parameters were significantly different as compared with their non-PS mTBI cohort, when the data were again combined across the white light and red light test conditions for the aforementioned reasons:

- Maximum diameter;
- Minimum diameter;
- Maximum dilation velocity;
- T50;
- T75.

With the blue light test condition, only one parameter was significantly different:

- 6PSPD.

Thus, those with PS in the mTBI group exhibited a larger pupillary diameter throughout the entire dynamic 6-s PLR profile, as well as a more rapid return to baseline diameter, as reflected in the faster initial peak dilation velocities, and related faster T50- and T75-times. Only the T50 parameter was common to both diagnostic groups.

**Figure 7.**
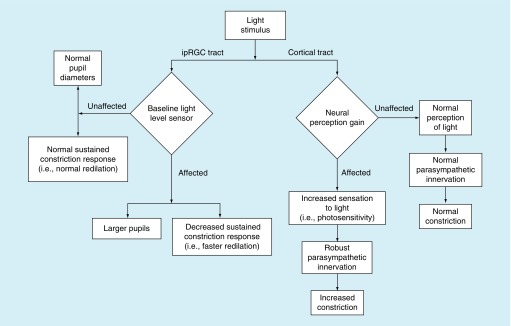
**Proposed photosensitivity flowchart to describe the possible mechanisms that may underlie the findings of the present study in both diagnostic groups.** Reprinted with permission from [11].

What are the possible neural substrates for PS in each population based on the above findings? It has been proposed that: there is a dysfunction in the baseline neural light sensor complex, likely residing in the suprachiasmic nucleus within the hypothalamus, as part of the ipRGC tract that is involved in the overall ambient light level and its perception, in those with PS in the mTBI population; and in contrast there is a dysfunction in the neural perceptual gain complex in the cortical tract in those with PS in the VN population. See Figure 7 schematic representation [11].

In the first case with mTBI, a normal/nondamaged baseline light sensor complex would result in both a normal pupil diameter and a normal sustained constriction response (i.e., normal redilation), and hence no PS. In contrast, if the sensor complex were damaged and rendered dysfunctional, it would produce a baseline pupil diameter offset, which could result in larger pupils and a decreased sustained constriction response (i.e., a faster redilation), both of which would allow more light to enter the eye, and likely giving rise, at least in part, to the sensation of PS.

In the second case for the normal population, a normal/nondamaged neural–perceptual gain complex would result in the normal perception of light, normal parasympathetic innervation and drive, and normal constriction, and hence no PS. In contrast, if this complex were damaged and rendered dysfunctional, it could produce an increased and abnormal perceptual sensation to the light (i.e., PS), and also related and more robust parasympathetic innervation, and hence increased constriction. Of interest, this more robust response was not found in those with mTBI and PS, possibly because their parasympathetic system is frequently adversely affected and effectively weakened by the brain trauma [5,11].

These findings have important clinical ramifications. For the first time, objective, noninvasive, rapid, vision-based, pupillary biomarkers for photosensitivity have now been discovered, quantified, and compared, as also briefly mentioned in question 2. This is valuable, as PS is a subjective perceptual phenomenon that could not, until recently, be confirmed objectively. Such information is critical for both the military and civilian populations, as it provides an unbiased means for evaluation and determination of PS in many important situations [27,28]. In the military, this would include fit-for-duty and return-to-duty standards, as well as possible related disability determination. In the civilian cohort, it would include worker's compensation determination, and social security disability and benefits determination, as well as return-to-play/work/learn standards for both adults and children. Furthermore, ROC analysis, as described earlier in question 2, appears to be valuable in evaluating the predictive power of a given parameter. For example, this combined ROC/objective pupillary recording approach could be used in a military or civilian hospital, objectively based vision screening for PS, perhaps at pre-deployment in the former case and for disability assessment in the latter case. These ideas are rapidly becoming a reality with advances in pupil testing, instrumentation and design, as well as more advanced and sensitive computer algorithms.

### Question 4: does refractive error influence pupillary responsivity?

Over the past decade or so, advances in development of objectively based, quantitative analysis of the PLR in mTBI [11,13–14,17–20] have received renewed interest, for reasons briefly mentioned in the Introduction section. Of particular interest to the clinician is the possible influence of refractive error on static and dynamic aspects of the pupil. The earlier findings with regard to the static (i.e., steady-state/baseline) maximum pupillary diameter were equivocal. Some clinicians reported that myopes tended to have larger pupillary diameters than either hyperopes or emmetropes [7,29], whereas others did not uncover any differences across refractive groups using more sophisticated laboratory measures [30–33]. Interestingly, neither the rationale nor possible mechanisms to support the idea of a refractive error-based influence on the pupil were considered.

To address these inconsistencies and gaps in our knowledge, an investigation was conducted [20] in both a VN group and in a matched one with a medical diagnosis of chronic mTBI over a wide range of static and dynamic parameters (as described earlier in ‘Static & dynamic pupillary light responses in mTBI: test protocols & instrumentation.’). There were seven pupillary parameters (maximum diameter, constriction latency, average constriction velocity, maximum/peak constriction velocity, average dilation velocity, maximum/peak dilation velocit and dynamic constriction amplitude), and four white light, flash stimulus conditions (dim pulse and step; bright pulse and step): thus, there were 28 test condition comparisons. Ages ranged from 21–60 years, and refractive errors ranged from -9 D to +1.25 D. None had either an APD or a pupillary opacity as assessed clinically. The response functions for each parameter (i.e., pupillary parameter value as a function refractive error) were fit with either a linear or Gaussian profile (i.e., normal distribution/bell-shaped) using root mean square analysis [34]. The root mean square analysis quantifies the difference between values predicted by a model (e.g., linear) and the observed data values, and furthermore seeks the best fit to minimize these differences, along with assessment of its statistical significance (p < 0.05).

Regarding the VN population:

- 21 out of the 28 test pupillary comparisons had significant fits, and thus exhibited a refractive-based response profile, with 20 out of 21 of the fits being Gaussian;
- The four most consistent pupillary parameters, with all having a Gaussian fit, were average constriction velocity, average dilation velocity, maximum/peak constriction velocity and dynamic response amplitude;
- The two test conditions demonstrating the most consistent fits (i.e., six out of seven parameters), with most being Gaussian, were for the bright pulse and dim step stimuli.

Regarding the mTBI population:

- Twenty-two out of 28 test comparisons had significant fits, and thus exhibited a refractive-based response profile, with 22 out of 22 being Gaussian;
- The four most consistent parameters, with all having a Gaussian fit, were average constriction velocity, maximum/peak constriction velocity, maximum/peak dilation velocity and maximum diameter; and
- All but the bright pulse test stimulus condition gave the most consistent fits (i.e., six out of seven parameters).

Interestingly, for the four test conditions and across both diagnostic groups, the latency parameter revealed lack of a significant fit with either the linear or Gaussian profiles. Thus, there was no influence of refractive error on the PLR latency.

**Figure 8.**
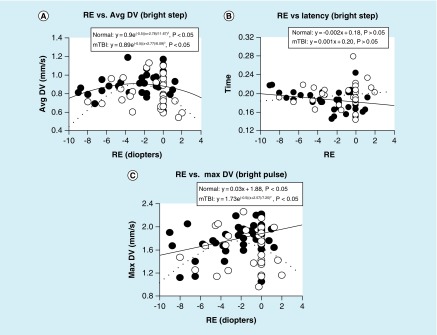
**Selected representative pupillary parameter profiles as a function of refractive error in diopters showing the three general response categories.** Data points represent the mean value for each subject. Closed circles represent the normals, and open circles represent those with mTBI. Solid lines represent the best fit for the normals, and dashed lines represent the best fit for those with mTBI. Time is in seconds. Avg DV: Average dilation velocity in mm/second; Max DV: Maximum dilation velocity in mm/second; RE: Refractive error.

Lastly, for both populations, the maxima of the Gaussian response profile ranged from approximately -2.3 to -4.9 D of myopia depending on the specific parameter (Figure 8). That is, over this myopic range, the response was maximum and approximately equal. Outside this range, the response profile dropped off, thus indicating that the pupillary parameter value was reduced relative to that found over the aforementioned optimal myopic range. For example, for peak pupillary constriction velocity, a critical and important diagnostic parameter, the value was reduced by about 20% in an 8D myope as compared with that of a 4D myope, in the normal population. Moreover, the response profile for the mTBI population was always reduced/depressed relative to, and frequently parallel with, that of the VN population. This is consistent with earlier dynamic, pupillary based, investigative findings in mTBI versus normals, when values were averaged *across* the range of refractive errors [11,13–14,17–19].

There are at least two possible mechanisms that might account for this finding: anatomical/biomechanical and pharmacological. In the former case, there are well-known differences in the size and/or shape of the pupillary aspects and surrounding anatomical structures in the anterior segment of the eye that have been found to be contingent upon an individual's refractive state (e.g., anterior chamber depth) [35]. Such architectural differences could result in a correlated, biomechanically based optimization of the dynamic response for a certain range of refractive errors. This notion should be investigated in the context of computer-based, biomechanical models (e.g., Matlab) of the dynamic pupillary system. In the latter case, we speculate that refractive-based, pharmacological differences may be involved. This is true for the human accommodative system where myopes may not have full access to the sympathetic system [36], and moreover the maximum accommodative amplitude is greatest for a 2D myope [37]. These ideas are consistent with the common finding of deficient sympathetic drive in those with mTBI [5,11]. Further investigations into this area are warranted.

The lack of relation between pupillary latency and refractive error is interesting. Latency likely reflects a predominantly neurosensory phenomenon, and not one of either a primary biomechanical or neuropharmocological nature. Hence, our latency finding is consistent with this notion, and furthermore supports our speculation regarding the other parameters having a primary biomechanical and/or pharmacological basis.

The clinical implications are quite clear and important. For most of the pupillary parameters assessed objectively and quantitatively in these two populations, refractive error magnitude may need to be factored into the diagnosis. For example, in a high myope (e.g., -7D) or hyperope (e.g., +2D), a pupillary parameter value slightly lower than the ‘norm’ found over the more common and optimal mid-myopic range would be expected, and in fact be considered normal. This information would be critical in the differential diagnosis, especially in either borderline cases or where the response variability is considerable. Further investigation using the latency parameter is warranted, as it was the only one that was refractive-error independent, and thus could have more general and simple usage in the future in the mTBI population.

## Conclusion

Based on the findings of the present review, the pupillary system may indeed provide a simple and non-invasive, objectively-based 'window' into the important and prevalent medical condition of mTBI, both with respect to documenting its presence, as well as the common elusive and bothersome symptom of photosensitivity. Such information will prove to be especially valuable in the clinical evaluation of the pupillary system in mTBI/TBI, as well as in other neurological conditions, which presently is relatively primitive when compared to the abilities of quantitative, automated pupillometry. Application of the ideas presented here should improve our understanding of the neurology of mTBI, as well as result in a higher level of clinical care.

## Future perspective

What holds for the future in the area of the objective assessment of the pupil in mTBI? We believe that there will be great advances and strides in a number of areas over the next 5–10 years.

### Rapid, objective & dynamic pupillary assessment

There will likely be several major inroads here. With current instrumentation, the PLR could be used in many settings requiring an immediate response regarding the possibility of having sustained a very early, acute-stage mTBI/concussion. This would include the sports sidelines/field/arena, military theater, emergency room and triage medical centers, and practices of sports medicine doctors and related professionals, as well as other possible types of health centers as the field advances. Related to this, and as mentioned earlier, it may become critical to assess separately the pupillary system over the acute, subacute and chronic phases post insult, as they may exhibit different abnormalities at each phase that would be diagnostic, as well as possibly also being prognostic indicators. Advances in future hardware technology and related computer algorithms will result in newer, less expensive and more sensitive types of instrumentation for pupillary assessment. For example, based on some of the most recent findings, portable pupillometers in the future will need to incorporate additional stimuli (e.g., red light, step profiles) to improve and broaden their diagnostic capabilities.

### Objective vision-based biomarkers

Based on earlier results [13,14], as well as the recent findings of Truong and Ciuffreda [11,17–20], there are several pupillary parameters that could serve as noninvasive, objectively based vision biomarkers for the presence of mTBI/concussion, as well as for PS in both the mTBI and VN populations. This could lead to the use of predeployment, vision screening/testing in the military to assess for either a pre-existing mTBI or for the presence of PS; for preseason vision screening/testing of mTBI/concussion at all levels of sports; pre-employment vision screening/testing for presence of a pre-existing mTBI/concussion in occupations in which the possibility of such an acquired brain injury has a relatively high probability and the workers are at risk, such as construction workers, police and prison guards, cab drivers in big cities, stuntmen and loggers; and for pre-incarceration vision screening/testing, as this population is very concussion prone (60–80%) [2]. The same holds true for victims of domestic violence [2].

### Correlates

It would be insightful to perform correlational analyses with other potential parameters and physiological systems that may also contain objective biomarkers for mTBI. These might include blood serum composition, spinal fluid, visually evoked potential latency and brain imaging voxel expression/strength, to name a few. This information would likely provide guidance (e.g., diagnostic or prognostic indicators) as to pervasiveness of the insult, as well as suggest neurophysiological linkages between the various systems and related neural networks with the pupillary pathway *per se*.

### Natural history changes

This is an area of great importance. However, very little research toward our understanding of the brain's naturally occurring neuroplasticity, its reaction to a traumatic brain event and the resultant biological ‘stress’ at all levels, including cellular (e.g., apoptosis) and molecular (e.g., altered calcium homeostatis) aspects, has been investigated. One should investigate the pupillary system, as well as related oculomotor systems (e.g., vergence) and their critical/high yield, abnormal parameters for mTBI (e.g., vergence peak velocity, near point of convergence) [2,38], and other nonoculomotor vision (e.g., visually evoked potential, eye-hand RT) and nonvision (e.g., attention, memory) systems [2–3,5,39].

### Objective swinging flashlight test

While the conventional, clinical swinging flashlight test is useful for detection of APD in particular, and will remain so, it has considerable limitations as described briefly earlier (e.g., resolution and sensitivity). Development of a simple, inexpensive, hand-held pupillometer that allows for accurate binocular recording and monocular/binocular light stimulation, as well as automated analysis and display, will likely be developed as its value becomes more evident to the medical and related professional communities (e.g., optometry and neurology). This type of device would be especially useful in vision screenings in third-world countries, remote settings such as small native Alaskan villages and nursing homes to improve diagnostic capabilities.

### Blast versus nonblast vision problems

An important question to address is the possibility of there being different visual deficits, and related unique visual problems and symptoms, for blast versus nonblast-induced TBI. Only one study made a direct comparison between these two groups related to vision. This involved a retrospective, clinical study (n = 500, US military personnel) that directly compared the visual aspect for each of the two etiologies [40]. Interestingly, when comparing those with a blast versus nonblast insult across the three postinjury stages (acute, subacute and chronic), few differences were found (eye pain, diplopia). However, they were only related to the postinjury stage and not the etiology. Unfortunately, the pupillary system was not assessed. However, as expected, there was the typical constellation of clinically based, binocular dysfunctions of high prevalence [2–4,41], such as a receded near point of convergence and high near vertical phoria, but they were equally prevalent in both groups. As the authors point out, this commonality of clinical signs and symptoms suggests that both the nonblast and blast mTBI populations can be diagnosed and treated in a similar manner, at least to a first approximation. However, the question remains if this would hold true using more sensitive and dynamic laboratory-based tests [42]. This might include assessment of the peak velocity and latency of the pupillary system, as well as the related vergence and accommodative oculomotor systems. In a related study, questionnaires were used to assess a range of areas (e.g., cognition), along with positron emission tomography brain imaging, in 12 warriors who sustained a pure blast mTBI versus 12 warriors who sustained a pure blunt mTBI [43]. Vision was not directly addressed. However, there were significant differences in attentional control, with correlated abnormalities noted in the right, parietal–frontal area of the brain involving attentional processing. Such general attentional disturbances are likely to be carried over to the visual domain [39,44].

### Pediatric mTBI vision problems

As in adults, the pediatric patient with mTBI (concussion) frequently exhibits a similar range of visual sequelae [45]. These typically include a host of oculomotor problems, such as convergence and accommodative deficits [46–50]. Many of these visual conditions have been documented to cause academic problems [51,52], if they persist once the child returns to the school environment (i.e., return-to-learn criteria) with its high visual demands [53]. Moreover, the child (e.g., a 5-year-old) with persistent and chronic visual and nonvisual problems (such as headache and nausea) may have a longer period of potential, adversely affected years than the adult (e.g., a 50 years old) with a recent mTBI. Hence, diagnosis and remediation are especially important in the child with mTBI. Unfortunately, due to their young age, a likely unreliable self reported history, and inaccurately reported symptoms, as well as the conventional relatively gross and subjective clinical vision assessment, the diagnosis, prognosis and possible therapeutic interventions may be difficult to ascertain [2,54].

However, the pupil holds promise in this youth population as a potential window to mTBI [2], especially with the relatively recent advent of simple, clinically based, objective, rapid and quantitative dynamic pupillometry [11,13–14,17–20], having as automated aspect [55]. This has been demonstrated in a series of recent papers in adults with mTBI [11,13–14,17–20]. Dynamic pupillometry could be used as a rapid means of attaining the above objective in children. One could assess for dynamic abnormalities (e.g., reduced PDV, increased latency), as well as possible pathological anisocoria and APD [55]. Developmental aspects of the pupil would have to be considered [11,56–57], especially regarding the normal small but consistent increases in baseline pupil, steady-state diameter with age (e.g., 5.34–6.27 from birth to 17 years of age) [58]. This is virgin territory for future clinical and laboratory exploration. Thus, the objectively based diagnosis of mTBI using pupillometry in the pediatric population appears to be bright.

**Executive summary**

**Basic PLR findings**

- The human pupillary light reflex (PLR) can respond to a 12 log-unit range of luminous intensity.
- In general, the rod and cone receptors control the initial phase of the PLR, whereas the intrinsically photosensitive retinal ganglion cell receptors control the latter phase.
- The parasympathetic system primarily controls pupillary constriction, whereas the sympathetic system primarily controls pupillary dilation.

**Static & dynamic interocular pupillary asymmetry in mild traumatic brain injury**

- There is no evidence of significant interocular pupillary asymmetry in the mTBI population, which is consistent with them having a postchiasmal brain insult.
- Physiologically appropriate interocular pupillary asymmetry in both the mild traumatic brain injury (mTBI) and normal populations was typically <0.66 mm.

**Dynamic PLR in mTBI**

- Responses were typically delayed, slowed, reduced and binocularly symmetrical in the mTBI population, as well as having a smaller initial baseline diameter.
- These findings suggest dysfunction of the afferent pupillary pathway, as well as the parasympathetic and sympathetic efferent pathways which are typically reduced in effectiveness in mTBI in general.
- Receiver operating characteristics analysis can be used to develop objectively based, vision screening criteria and resultant biomarkers for the presence of mTBI.

**PLR & photosensitivity in mTBI**

- Several PLR parameters discriminated between those with versus without photosensitivity (PS) in both the mTBI and visually normal (VN) populations.
- In mTBI, the PS is believed to be due to dysfunction in the baseline neural sensor, likely residing in the suprachiasmal nucleus and hypothalamus.
- In VN, the PS is believed to be due to dysfunction in the perceptual–gain complex in the cortical tract.
- Receiver operating characteristics analysis combined with dynamic pupillometry can be used to provide objective biomarkers for the presence of PS in both the mTBI and the VN populations.

**PLR & refractive state**

- Most pupillary parameters exhibited refractive-dependent response profiles.
- Pupillary responsivity was maximal in the myopic range of 2.3–4.9 diopters, with it being reduced outside this range.
- This refractive dependence for most parameters may be related to ocular biomechanical and/or pharmacological factors.
- Only latency was not influenced by refractive error.

**Conclusion**

- The PLR in mTBI is an important vision-based, physiological system to understand and investigate both in the clinic and laboratory in the mTBI population.
- Instrumentation in the future will be developed to exploit the PLR diagnostically in both the military and civilian populations with mTBI and/or PS to improve patient care.

## References

[1] Blennow K, Brody DL, Kochanek PM (2016). Traumatic brain injuries. *Nat. Rev. Dis. Primer*.

[2] Ciuffreda KJ, Ludlam DL, Yadav NK, Thiagarajan P, Myron Y (2016). Traumatic brain injury: visual consequences, diagnosis, and treatment. *Advances in Ophthalmology and Optometry.*.

[3] Suchoff IB, Ciuffreda KJ, Kapoor N (2001). *Visual and Vestibular Consequences of Acquired Brain Injury.*.

[4] Thiagarajan P (2013). Oculomotor rehabilitation for reading dysfunction in mild traumatic brain injury. http://hdl.handle.net/1951/60357.

[5] Zasler ND, Katz DI, Zafonte RD (2012). *Brain Injury Medicine: Principles and Practice*.

[6] Baylis GC, Baylis LL (1997). Deficit in figure-ground segmentation following closed head injury. *Neuropsychologia*.

[7] Zinn KM, Charles C (1972). *The Pupil.*.

[8] Kardon RH, Levin LA, Nilsson SFV, Ver Hoeve J, Wu SM (2011). Regulation of light through the pupil. *Adler's Physiology of the Eye*.

[9] Park JC, Moura AL, Raza AS, Rhee DW, Kardon RH, Hood DC (2011). Toward a clinical protocol for assessing rod, cone and melanopsin contributions to the human pupil response. *Invest. Ophthalmol. Vis. Sci.*.

[10] Ciuffreda KJ, Benjamin WJ (2006). Accommodation, the pupil, and presbyopia. *Borish's Clinical Refraction (2nd Edition)*.

[11] Truong JQ (2016). Mild traumatic brain injury (mTBI) and photosensitivity: objective pupillometric findings. http://hdl.handle.net/1951/67743.

[12] Loewenfeld IE, Lowenstein O (1993). *The Pupil: Anatomy, Physiology, and Clinical Applications*.

[13] Capó-Aponte JE, Urosevich TG, Walsh DV, Temme LA, Tarbett AK (2013). Pupillary light reflex as an objective biomarker for early identification of blast-induced mTBI. *J. Spine*.

[14] Thiagarajan P, Cuiffreda KJ (2015). Pupillary responses to light in chronic non-blast-induced mTBI. *Brain Inj.*.

[15] Ellis CJ (1981). The pupillary light reflex in normal subjects. *Br. J. Ophthalmol.*.

[16] NeuroOptics http://www.neuroptics.com.

[17] Truong JQ, Ciuffreda KJ (2016). Comparison of pupillary dynamics to light in the mild traumatic brain injury (mTBI) and normal populations. *Brain Inj.*.

[18] Truong JQ, Ciuffreda KJ (2016). Quantifying pupillary asymmetry through objective binocular pupillometry in the normal and mild traumatic brain injury (mTBI) populations. *Brain Inj.*.

[19] Truong JQ, Ciuffreda KJ (2016). Objective pupillary correlates of photosensitivity in the normal and mild traumatic brain injury populations. *Mil. Med.*.

[20] Truong JQ, Joshi NR, Ciuffreda KJ (2016). Influence of refractive error on pupillary dynamics in the normal and mild traumatic brain injury (mTBI) populations. *J. Optom.*.

[21] Loewenfeld I (1977). “Simple central” anisocoria: a common condition, seldom recognized. *Trans. Sect. Ophthalmol. Am. Acad. Ophthalmol. Otolaryngol.*.

[22] Lam BL, Thompson HS, Corbett JJ (1987). The prevalence of simple anisocoria. *Am. J. Ophthalmol.*.

[23] Metz CE (1978). Basic principles of ROC analysis. *Semin. Nucl. Med.*.

[24] Von Noorden GK, Maumenee AE (1967). *Atlas of Strabismus*.

[25] Fimreite V, Ciuffreda KJ, Yadav NK (2015). Effects of luminance on the visually-evoked potential in visually-normal individuals and in mTBI/concussion. *Brain Inj.*.

[26] Truong JQ, Ciuffreda KJ, Han MHE, Suchoff IB (2014). Photosensitivity in mild traumatic brain injury (mTBI): a retrospective analysis. *Brain Inj.*.

[27] Brooks N, McKinlay W, Symington C, Beattie A, Campsie L (1987). Return to work within the first seven years of severe head injury. *Brain Inj.*.

[28] Cantu RC (1998). Return to play guidelines after a head injury. *Clin. Sports Med.*.

[29] Hirsch MJ, Weymouth FW (1949). Pupil size in ametropia. *J. Appl. Physiol.*.

[30] Winn B, Whitaker D, Elliott DB, Phillips NJ (1994). Factors affecting light-adapted pupil size in normal human subjects. *Invest. Ophthalmol. Vis. Sci.*.

[31] Cakmak HB, Cagil N, Simavlı H, Duzen B, Simsek S (2010). Refractive error may influence mesopic pupil size. *Curr. Eye Res.*.

[32] Orr JB, Seidel D, Day M, Gray LS (2015). Is pupil diameter influenced by refractive error?. *Optom. Vis. Sci.*.

[33] Adhikari P, Pearson CA, Anderson AM, Zele A, Feigl B (2015). Effect of age and refractive error on the melanopsin mediated post-illumination pupil response (PIPR). *Sci. Rep.*.

[34] Applegate RA, Ballentine C, Gross H, Sarver EJ, Sarver CA (2003). Visual acuity as a function of zernike mode and level of root mean square error. *Optom. Vis. Sci.*.

[35] Gilmartin B, Nagra M, Logan NS (2013). Shape of the posterior vitreous chamber in human emmetropia and myopia. *Invest. Ophthalmol. Vis. Sci.*.

[36] Gilmartin B, Rosenfield M (1998). Autonomic correlates of near-vision in emmetropia and myopia. *Myopia and Near Work*.

[37] McBrien NA, Millodot M (1986). Amplitude of accommodation and refractive error. *Invest. Ophthalmol. Vis. Sci.*.

[38] Thiagarajan P, Ciuffreda KJ (2013). Effect of oculomotor rehabilitation on vergence responsivity in mild traumatic brain injury. *JRRD*.

[39] Yadav NK, Thiagarajan P, Ciuffreda KJ (2014). Effect of oculomotor vision rehabilitation on the visual-evoked potential and visual attention in mild traumatic brain injury. *Brain Inj.*.

[40] Capo-Aponte JE, Jorgensen-Wagers KL, Sosa JA (2017). Visual dysfunctions at different stages after blast and non-blast mild traumatic brain injury. *Optom. Vis. Sci.*.

[41] Ciuffreda KJ (2016). *Compendium of Works on Visual Rehabilitation*.

[42] Ciuffreda KJ, Ludlum DP, Thiagarajan P, Yadav NK, Capo-Aponte J (2014). Proposed objective visual system biomarkers for mild traumatic brain injury. *Mil. Med.*.

[43] Mendez ME, Owens EM, Reza Berenji G, Peppers DC, Liang LJ, Licht EA (2013). Mild traumatic brain injury from blast vs. blunt forces: post-concussion consequences and functional neuroimaging. *Neurorehabilitation*.

[44] Yadav NK, Ciuffreda KJ (2014). Objective assessment of visual attention in mild traumatic brain injury (mTBI) using visual-evoked potentials (VEP). *Brain Inj.*.

[45] Suter PS, Harvey LH (2011). *Vision Rehabilitation*.

[46] Bodack MI (2010). Pediatric acquired brain injury. *Optometry*.

[47] Grubenhoff JA, Kirkwood MW, Deakyne S, Wathen J (2011). Detailed concussion symptom analysis in a paediatric ED population. *Brain Inj.*.

[48] Ellis MJ, Cordingley D, Vis S, Reimer K, Leiter J, Russell K (2015). Vestibulo-ocular dysfunction in pediatric sports-related concussion. *J. Neurosurg. Pediatr.*.

[49] Master CL, Scheiman M, Gallaway M (2016). Vision diagnoses are common after concussion in adolescents. *Clin. Pediatr. (Phila)*.

[50] Ellis MJ, Cordingley DM, Vis S, Reimer KM, Leiter J, Russell K (2017). Clinical predictors of vestibulo-ocular dysfunction in pediatric sports-related concussion. *J. Neurosurg. Pediatr.*.

[51] Russell K, Hutchinson MG, Selci E, Leiter J, Chateau D, Ellis MJ (2016). Academic outcomes in high-school students after a concussion: a retrospective population-based study. *PLoS ONE*.

[52] Swanson MW, Weise KK, Dreer LE (2017). Academic difficulty and vision symptoms in children with concussion. *Optom. Vis. Sci.*.

[53] Baker JG, Rieger BP, McAvoy K (2014). Principles for return to learn after concussion. *Inj. J. Clin. Pract.*.

[54] Barnett BP, Singman EL (2015). Vision concerns after mild traumatic brain injury. *Curr. Treat. Opt. Neurol.*.

[55] Singman EL (2013). Automating the assessment of visual dysfunction after traumatic brain injury. *Med. Instrument.*.

[56] MacLachlan C, Howland HC (2002). Normal values and standard deviations for pupil diameter and interpupillary distance in subjects aged 1 month to 19 years. *Ophthalm. Physiol. Opt.*.

[57] Boev AN, Fountas KN, Karampelas J (2005). Quantitative pupillometry: normative data in healthy pediatric volunteers. *J. Neurosurg.*.

[58] Silbert J, Matta N, Tian J, Singman EL, Silbert DI (2013). Pupil size and anisocoria in children measured by the plusoptiX photoscreener. *J. AAPOS*.

