# Supplementary figures and images for: Understanding the effects of mild traumatic brain injury on the pupillary light reflex
Source: Concussion. 2017 Aug 3;2(3):CNC36. doi: 10.2217/cnc-2016-0029 (PMC6094691; doi:10.2217/cnc-2016-0029)

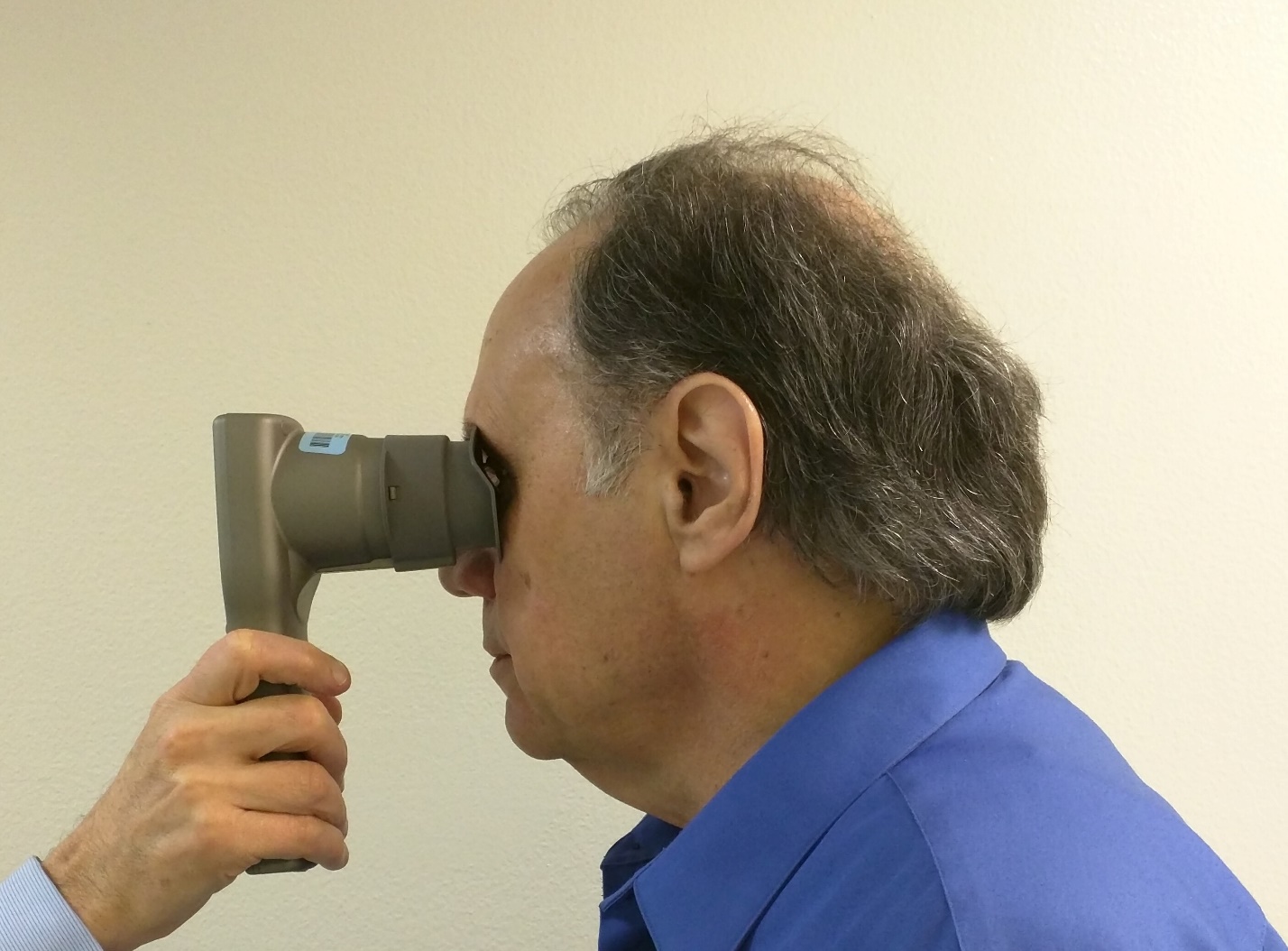

Supplement: Supplementary file 1 [file cnc-02-36-s1.jpg]
